# A Non-Invasive Method of Quantifying Pancreatic Volume in Mice Using Micro-MRI

**DOI:** 10.1371/journal.pone.0092263

**Published:** 2014-03-18

**Authors:** Jose L. Paredes, Abrahim I. Orabi, Taimur Ahmad, Iman Benbourenane, Kimimasa Tobita, Sameh Tadros, Kyongtae T. Bae, Sohail Z. Husain

**Affiliations:** 1 Department of Pediatrics, Children's Hospital of Pittsburgh of UPMC and the University of Pittsburgh, Pittsburgh, Pennsylvania, United States of America; 2 Department of Developmental Biology, Children's Hospital of Pittsburgh of UPMC and the University of Pittsburgh, Pittsburgh, Pennsylvania, United States of America; 3 Department of Radiology, Children's Hospital of Pittsburgh of UPMC and the University of Pittsburgh, Pittsburgh, Pennsylvania, United States of America; Garvan Institute of Medical Research, Australia

## Abstract

In experimental models of pancreatic growth and recovery, changes in pancreatic size are assessed by euthanizing a large cohort of animals at varying time points and measuring organ mass. However, to ascertain this information in clinical practice, patients with pancreatic disorders routinely undergo non-invasive cross-sectional imaging of the pancreas using magnetic resonance imaging (MRI) or computed tomography (CT). The aim of the current study was to develop a thin-sliced, optimized sequence protocol using a high field MRI to accurately calculate pancreatic volumes in the most common experimental animal, the mouse. Using a 7 Telsa Bruker micro-MRI system, we performed abdominal imaging in whole-fixed mice in three standard planes: axial, sagittal, and coronal. The contour of the pancreas was traced using Vitrea software and then transformed into a 3-dimensional (3D) reconstruction, from which volumetric measurements were calculated. Images were optimized using heart perfusion-fixation, T1 sequence analysis, and 0.2 to 0.4 mm thick slices. As proof of principle, increases in pancreatic volume among mice of different ages correlated tightly with increasing body weight. In summary, this is the first study to measure pancreatic volumes in mice, using a high field 7 Tesla micro-MRI and a thin-sliced, optimized sequence protocol. We anticipate that micro-MRI will improve the ability to non-invasively quantify changes in pancreatic size and will dramatically reduce the number of animals required to serially assess pancreatic growth and recovery.

## Introduction

Pancreas size is a key parameter that is used in the experimental setting to assess pancreatic growth, development, and recovery following injury [1], [2], [3], [4], [5]. Although many studies in pancreas development and regeneration use mouse models that exploit sophisticated transgenic technology, most of these studies only qualitatively describe changes in pancreatic size or are forced to weigh out the pancreas *ex vivo*. The challenge is that quantifying the dynamic flux in pancreas size necessitates that a large cohort of animals be euthanized at varying time points. There is also the technical difficulty, particularly in the mouse, of accurately identifying the entire pancreas, because it is small, soft in texture, and juxtaposed with the stomach, spleen, left kidney, and intestine [6], [7].

In clinical practice, however, patients with pancreatitis or pancreatic insufficiency routinely undergo cross-sectional imaging to assess for pancreatic changes. Although there are several published protocols in humans to calculate pancreatic volume using CT [8], [9] and MRI [10], [11], [12], [13], [14], methods in animals models are limited to large animals [10]. In this study, we optimized a method for accurately quantifying pancreatic volume in mice using a 7 Tesla micro-MRI and a thin-sliced RARE sequence protocol.

## Materials and Methods

### Reagents and animals

All reagents were purchased from Sigma-Aldrich (St. Louis, MO) unless otherwise stated. Male Swiss Webster mice (Charles River, Wilmington, MA) weighing 15 to 70 g and from 21 days to 9 months of age were fed standard laboratory chow with free access to water. All animal experiments were performed using a protocol approved by the University of Pittsburgh Institutional Animal Care and Use Committee.

### *In vivo* perfusion fixation

Mice were euthanized by CO_2_ asphyxiation, followed by cervical dislocation. Heart perfusion fixation was performed with a procedure previously described [15]. Briefly, the skin covering the thorax and abdomen was removed, and a right lateral thoracotomy was performed. A 27G butterfly needle was inserted into the left ventricle and held in place with a fine tip hemostat. The bulk of the circulating blood was drained by making a small incision in the right atrium. Ten ml of phosphate buffered saline was infused through the left ventricle for 5 min, followed by infusion of 4% paraformaldehyde (PFA) until clear fluid was observed in the right atrium and the ears and nose turned pale. To maximize exposure of the fixative to the target region, 3 ml of 4% PFA was injected into the abdominal cavity, and the animal was gently rotated for 5 min. Thereafter, 4 small incisions were made into the abdominal cavity, and the whole mouse was immersed into a 50 ml conical tube containing 4% PFA for at least 3 days.

### Micro-MRI setup

The whole-fixed mouse was transferred to a dry 50 ml conical tube, which was then secured to a micro-MRI cradle and advanced into the magnet (7 Tesla, Bruker BioSpec 70/30 USR, Bruker BioSpin Corporation Billerica, MA). An initial tri-pilot scan protocol was used to target in on the abdomen. A second more detailed tri-pilot multi-scan was performed to identify the location of the pancreas. Subsequently, various sequence protocols using ParaVision Acquisition 5.1 software were tested (Table 1).

**Table 1:** MRI sequence protocols and some of their key differences.

Sequence	Weighting	TE (ms)	TR (ms)	Flip angle	Field of view mmXmm	Technique
RARE (Rapid Acquisition with Relaxation Enhancement) with Fat Saturation	T1	7.5	1300	180	40X40	Spin echo, fast imaging
FISP (Fast imaging with Steady State Precession)	T2/T1	4	8	15	60X60	Gradient recalled echo
FLASH (Fast Low Angle Shoot) with Fat Saturation	T1	5.4	350	40	40X40	Gradient recalled echo
RARE-INV-REC with Fat Saturation	T1	7.5	3200	180	40X40	Spin echo, fast imaging with inversion recovery
TURBO RARE with Fat Saturation	T2	45	1500	180	40X40	Spin echo, fast imaging

TE, Echo Time; TR, Repetition Time. The matrix size for each of the sequences was 256X19.

### MRI tracing and volume calculation

Three sets of images were obtained from three orthogonal planes: axial, sagittal, and coronal. To calculate pancreas volume, the pancreas was first outlined in each axial image, primarily based on its anatomical location relative to adjacent organs. The spleen and superior aspect of the left kidney were used to identify the tail of the pancreas, and the stomach and intestine served as landmarks for the head and body of the pancreas. Ambiguities in outlining the pancreas in the axial plane were cross-checked by outlining the pancreas in corresponding sagittal and coronal planes. Discrete areas of intra-pancreatic fat, including areas circumscribed by pancreatic parenchyma, were excluded from the tracings. Pancreatic borders were outlined as tightly as possible, including along areas of fissures. From these traces, a 3D reconstruction of the pancreas was generated using the Vitrea Core software (Toshiba Medical Systems, Minnetonka, MN). Briefly, outlined pixels on cross-sections were converted into volumetric pixel units (voxels) based on slice thickness. Voxel volumes over the entire depth of the pancreatic slices were integrated to output a single pancreatic volume for each animal.

### Statistical analysis

Results were expressed as mean + standard deviation unless otherwise stated. Statistical significance between 2 groups was determined using a Student's t-test and for more than 2 groups, a one way ANOVA was used. A Pearson correlation coefficient was used to assess the degree to which two variables are related. Statistical significance was defined as a P value ≤0.05.

## Results

### Imaging the mouse pancreas and generating pancreatic volumes

**Figure 1:**
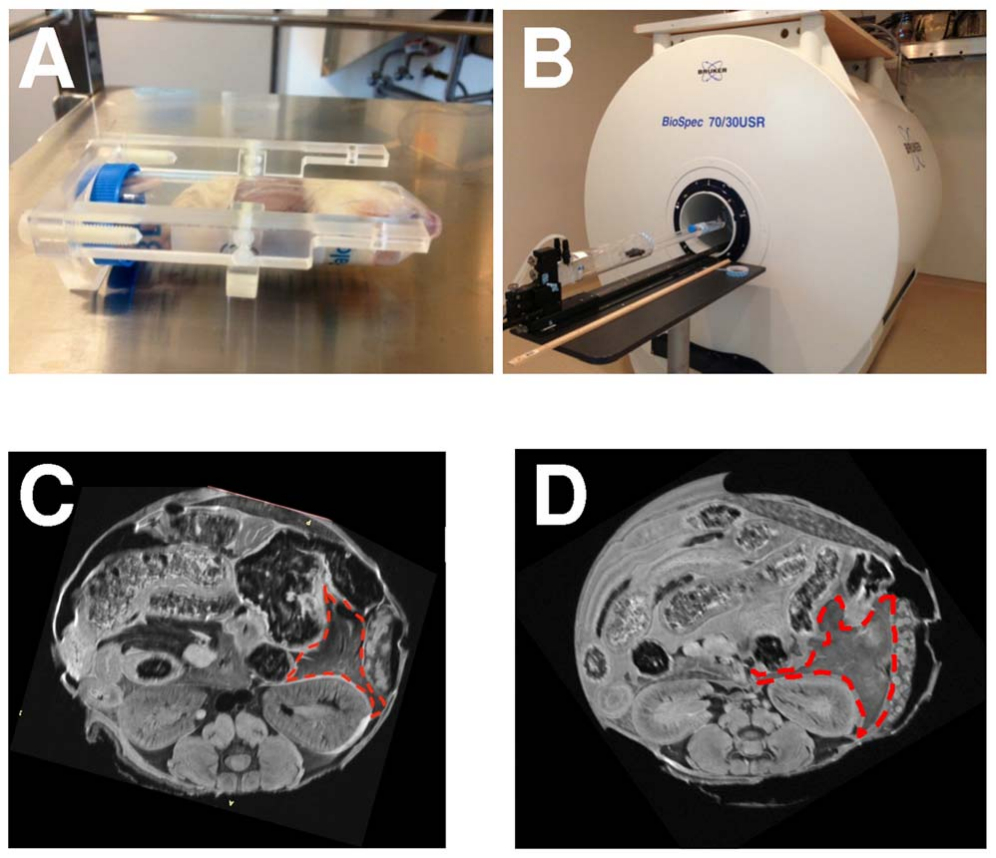
Preparation of the mouse for MRI. In these studies, whole-fixed mice were (A) placed in a conical tube and (B) inserted into a Bruker 7 Tesla micro-MRI. (C) Compared to immersion fixation, (D) *in vivo* perfusion fixation yielded a more homogenous pancreatic MRI signal (red outline).

**Figure 2:**
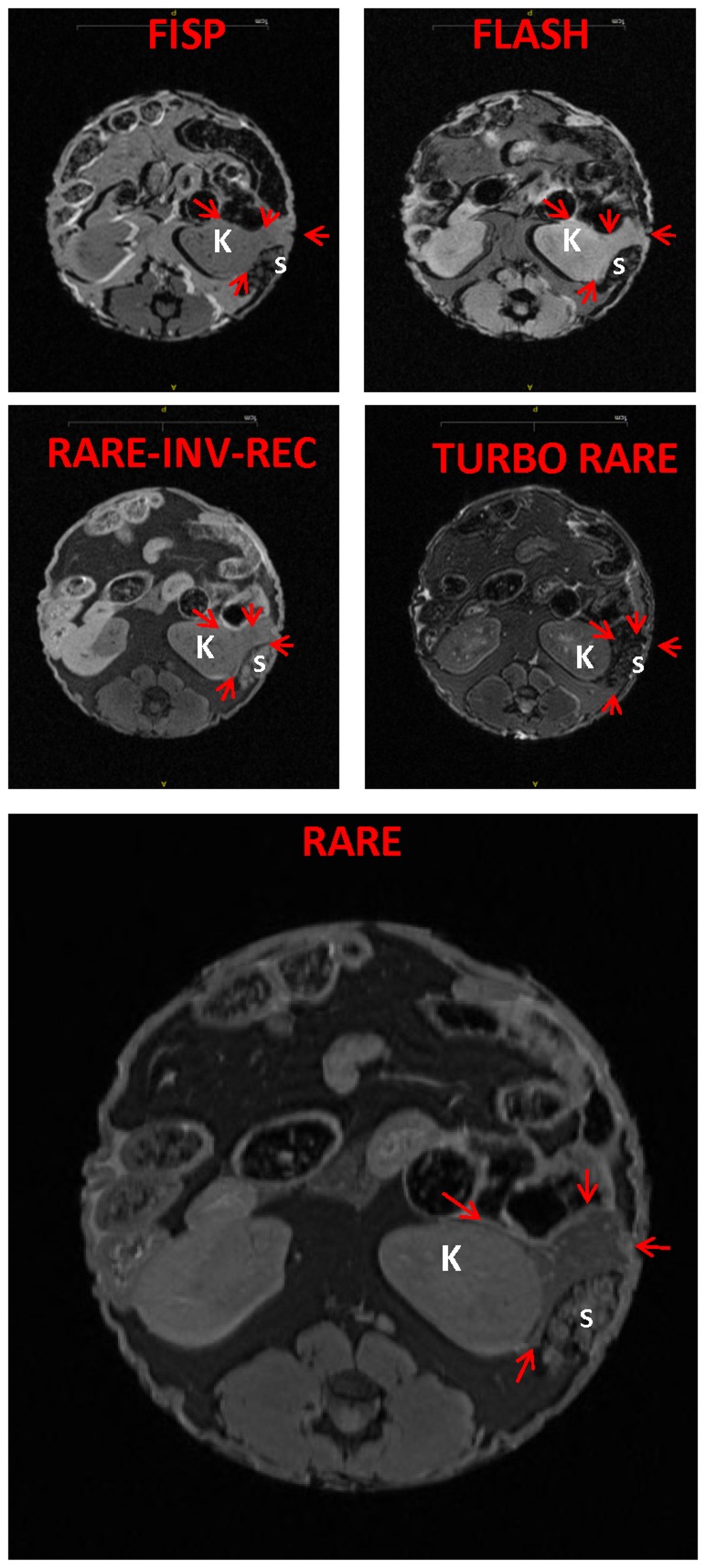
RARE is superior to other sequence formats in delineating the pancreas. Representative slices of the various sequence protocols demonstrate that RARE sequence provides the best delineation of the pancreas from adjacent organs. The arrows point to the pancreas. S, spleen; K, kidney.

To establish a proof of principle for imaging the mouse pancreas, the pancreas was measured *in situ* within the whole-fixed mouse (Fig. 1). An immersion fixation protocol was initially used. However, with this technique the pancreas on MRI cross-sections had a heterogeneous pattern, which indicated incomplete fixation (Fig. 1c). Thus *in vivo* heart perfusion was subsequently used, as previously described[15], and yielded a more homogeneous pancreatic signal (Fig. 1d). There are several sequence protocols for MRI [16], and they differ in multiple factors including T1- or T2-weighting, acquisition time, flip angle, and field of view (Table 1). RARE (Rapid Acquisition with Relaxation Enhancement) provided the best contrast between the pancreas and adjacent organs (including the kidney and spleen) and soft tissues (Fig. 2).

### Identifying a reliable sequence protocol and adequate slice thickness

**Figure 3:**
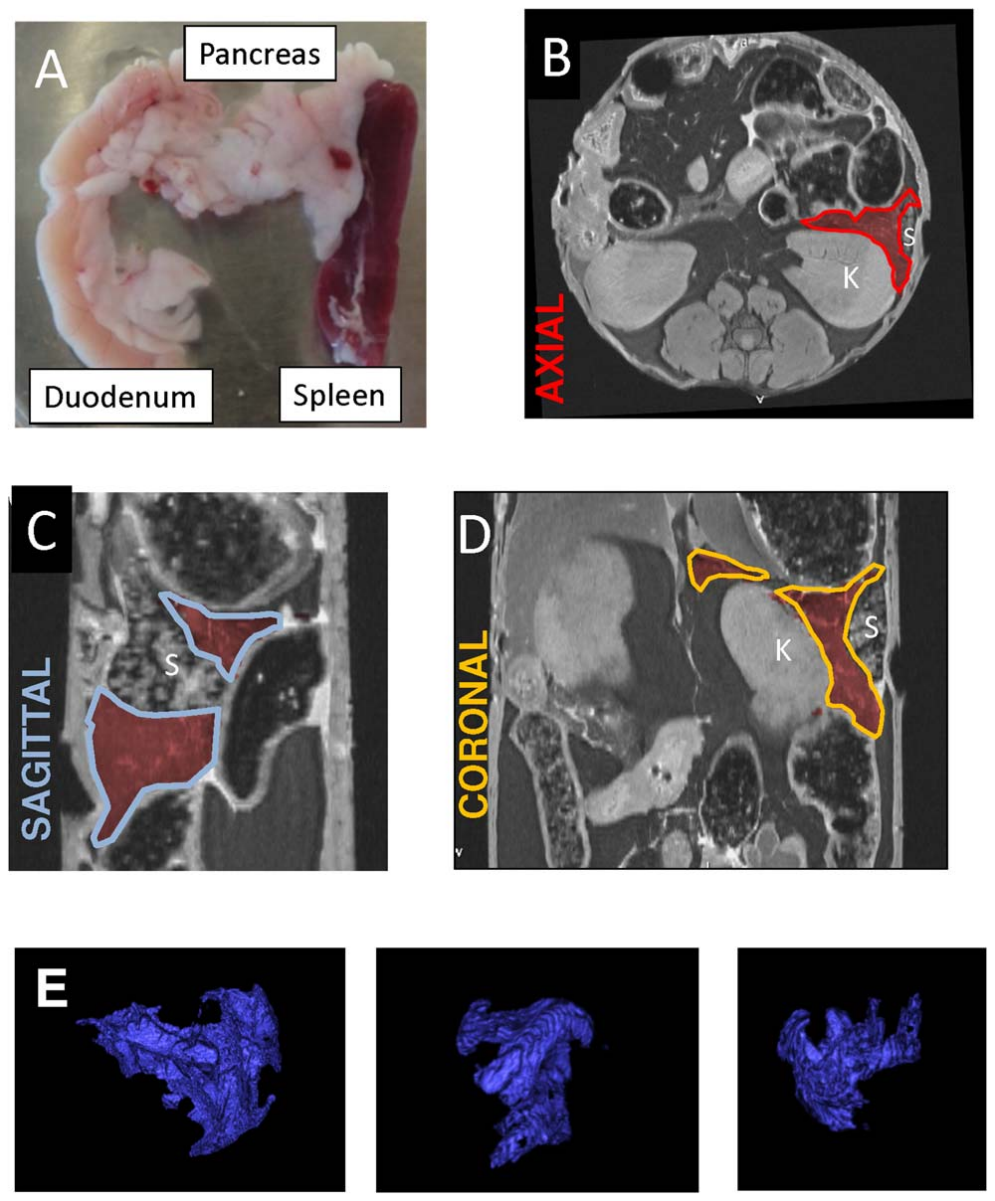
Method for generating a 3D reconstruction of the mouse pancreas. (A) Gross dissection of the pancreas with its adjoining organs. The pancreas was traced in each (B) axial image and, for further delineation, tracings were cross-checked, as necessary, using (C) sagittal and (D) coronal planes. (E) Three representative 3D reconstructions of the pancreas, generated using these tracings, are shown.

Using a RARE sequence, the mouse abdomen was imaged (Fig. 3). As a standard, the axial plane was chosen to manually trace the pancreas, and the adjacent organs were used as crucial landmarks. Small but distinct intrusions of peri-pancreatic fat, inter-digitated within areas of pancreatic parenchyma, was easily excluded from the tracings using a fat saturation protocol. Similarly, circumscribed areas of fat were also excluded from regions of interest. Peri-pancreatic and occasional intra-pancreatic lymph nodes were also avoided. Using the thinnest available slice thickness of 0.2 mm, there were 30 to 40 slices containing pancreatic tissue. From the slices, only 3 to 5 slices contained indiscrete pancreatic borders. In these cases, the pancreas was traced from corresponding sagittal and coronal planes. The line highlight tool was carried back to the axial plane in order to confirm the pancreatic border. A 3D reconstruction of the pancreas was generated (Fig. 3e), and pancreatic volume was calculated from the Vitrea software by integrating voxel units. Using this method of tracings to calculate mouse pancreatic volume, the inter-observer variability (for JLP) was 1.42% and the intra-observer variability (between JLP and IB) was 1.72%. The findings suggest that the method for pancreatic volume calculations is reliable.

**Figure 4:**
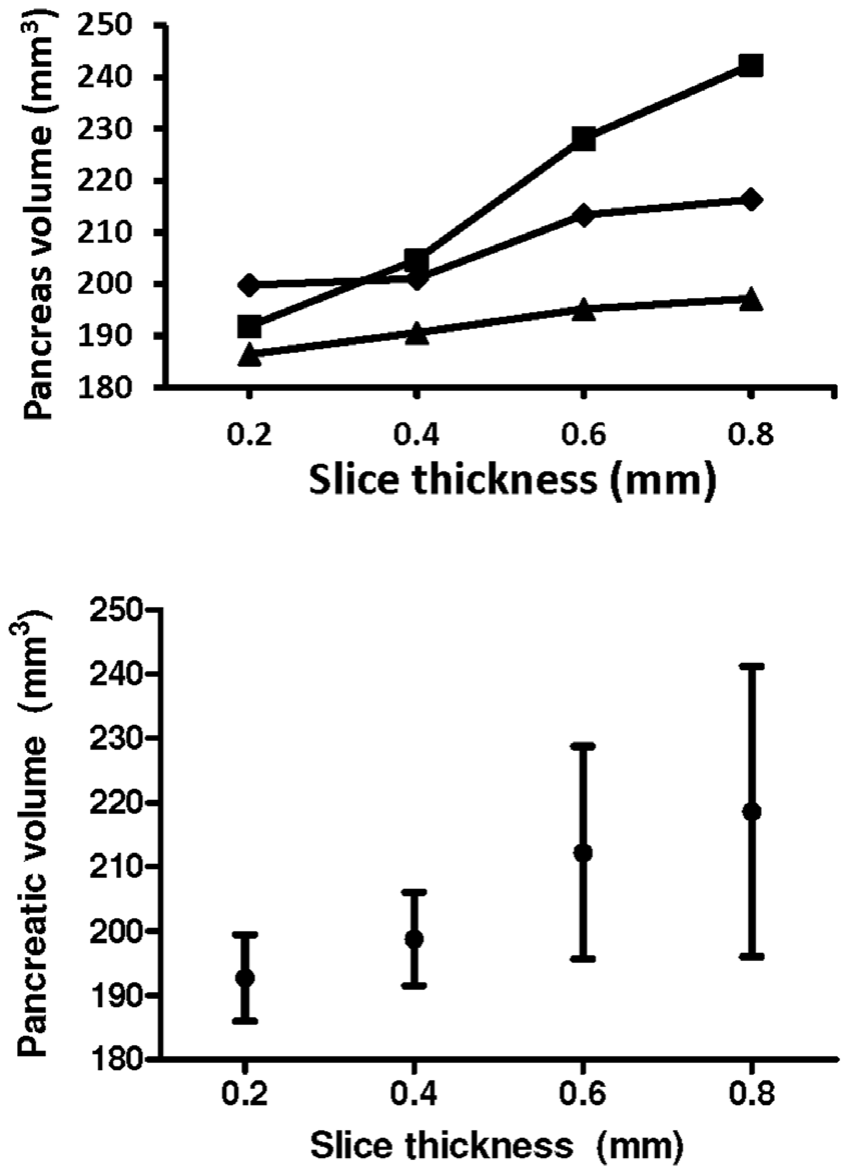
Thin cross-sectional slices are necessary to obtain accurate mouse pancreatic volume measurements. First, pancreatic volumes were calculated from the thinnest cross-sectional slice (0.2 mm; as described in the Methods). Next, volume calculations were also performed by skipping slices. (A) Volume calculations from 3 individual mice with increasing slice thickness. (B) Mean volumes ±1 standard deviation (for the 3 mice) demonstrate that volumes calculated from slices greater than 0.4 mm were substantially higher than the 0.2 mm “gold standard” and had higher variance.

To determine the minimum slice thickness necessary to derive pancreatic volumes similar to the thinnest 0.2 mm slice “gold standard,” volumetric calculations were performed at 0.4 mm, 0.6 mm, and 0.8 mm slice thickness in 3 mice of 9 months age (Fig. 4). The mean pancreatic volume with a 0.2 mm slice was 192.7 mm^3^±6.7 mm^3^. There was only a 3.2% difference in volumes with a 0.4 mm slice, and the variance (i.e. 1 standard deviation) was similar at 7.3 mm^3^. However, there was a 10.1% and 13.4% difference in volumes with 0.6 mm and 0.8 mm slice thickness, respectively, and they had high variance (16.5 mm^3^ and 22.6 mm^3^, respectively). These results indicate that slices of 0.4 mm or less are necessary to reliably image the mouse pancreas with our current sequence parameters.

### Confirming accuracy of small volume determinations by MRI and assessing pancreatic growth

**Figure 5:**
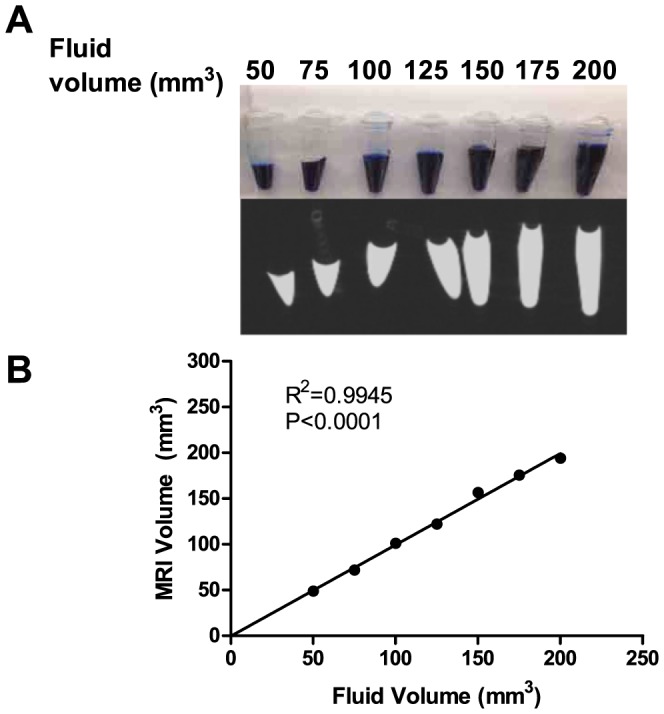
Micro-MRI can accurately measure small volumes in phantom tubes. (A) Known volumes of gadolinium contrast, ranging from 50 to 200 mm^3^, with 25 mm^3^ increments, were loaded into small conical tubes (top row) and imaged using an optimized MRI protocol (bottom row). (B) There was a tight correlation between known and MRI-measured volumes.

Volume displacement measurements of the dissected pancreas, used as a gold standard to compare MRI-generated pancreatic volumes in a recent study in pigs [10], for example, were difficult to obtain due to the small volume of the mouse pancreas (i.e. 100–200 mm^3^) and the sticky, amorphous nature of the organ. Thus strict accuracy of pancreatic volumes measurements by MRI with a paired gold standard could not be assessed in the mouse. Nonetheless, to confirm accuracy of detecting such small volumes by our micro-MRI methods, imaging of phantom tubes were performed (Fig. 5). MRI-measured volumes of gadolinium contrast-enhanced fluid within these tubes highly correlated with actual filling volumes (50–200 mm^3^; R2 = 0.9945; P<0.0001), suggesting that micro-MRI can accurately measure small volumes in the range of mouse pancreatic volumes (Fig. 5).

**Figure 6:**
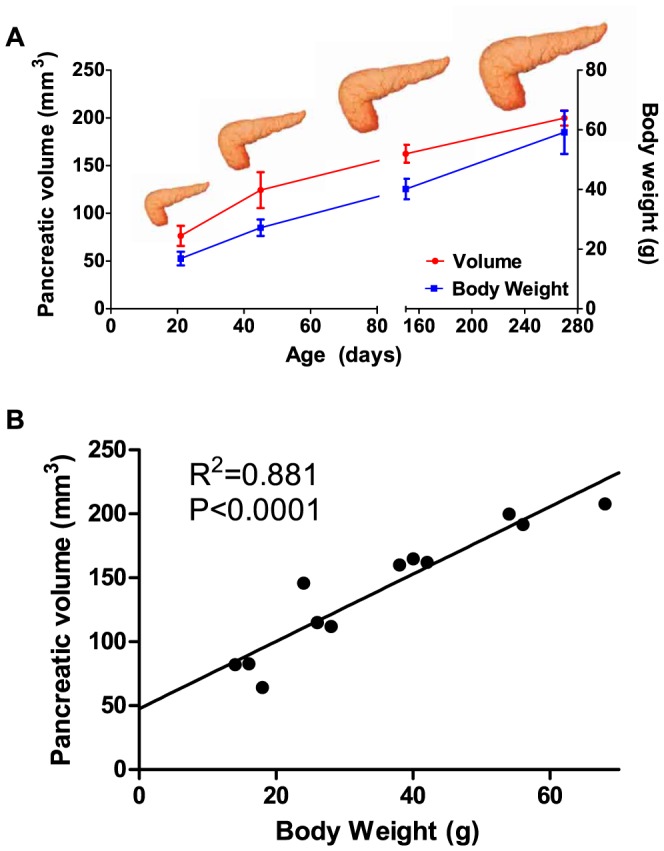
Micro-MRI can accurately measure increases in pancreatic volume with advancing age. (A) Changes in pancreatic volume (in red) correlate with body weight (in blue). (n = 3 mice per age group). *, p<0.05, using a one way ANOVA. (B) Scatter plot demonstrating that pancreatic volume correlates tightly with body weight.

To test the ability of the optimized MRI protocol to differentiate *in situ* differences in pancreatic volume, we next examined growth of the pancreas with advancing age (Fig. 6). We imaged mice that were available from the period of weaning (21 days old) to older retired breeders (280 days old). Overall, there was a 161.4% growth of the pancreas between 21 and 280 day old mice. Between 21 and 42 days of life, there was a 62.7% increase in pancreatic volume, representing a rapid rate of growth of 2.28 mm^3^/day, whereas between 42 and 280 days, there was a 60.7% increase, with a slower growth rate of 0.32 mm^3^/day. Pancreatic volume tightly correlated with body weight (R^2^ = 0.881, P<0.0001). The results indicate that MRI is sensitive enough to detect the small changes in pancreatic volume with advancing mouse age.

## Discussion

To our knowledge, this is the first study to non-invasively measure pancreatic volumes in mice, using a high field 7 Tesla micro-MRI and a thin-sliced, optimized sequence protocol. Most studies examining changes in pancreatic growth have been primarily assessed in a qualitative fashion [1], [2], whereas others have measured the weight of the pancreas [3], [4]. Bonner-Weir et al. estimated pancreatic volumes from mouse pancreas fragments by performing morphometric measurements from multiple histological sections [5]. However, a large number of animals are required to serially measure growth because they need to be euthanized at each time point. Further, the assays are tedious. The advantages of calculating pancreatic volume by MRI are that the method is non-invasive, thus reducing animal usage, and the same animal can now be tracked over time [17]. Although there are several imaging modalities, MRI is ideal for reliably measuring such small volumes as the mouse pancreas. Ultrasound is operator-dependent and this suffers from inter-observer variability. Among cross-sectional imaging modalities, MRI provides better soft tissue contrast than computed tomography (CT) and has better spatial resolution than positive emission tomography (PET). Nonetheless, there are several studies with established CT protocols for measuring pancreatic volume in humans [8], [9], [11].

Even with MRI, however, there are inherent challenges for mouse imaging. Most MRI studies that measured pancreatic volume in humans have used a 1.5 to 3 Tesla magnet and 5–10 mm thick slices [10], [12], [13], [14]. However, the mouse pancreas is about 500 times smaller in volume than the human pancreas, and thus MRIs with higher spatial resolution are necessary. Another issue is that the mouse pancreas is more amorphous than the human pancreas. Whereas the human pancreas is simply banana-shaped [18], the mouse pancreas is splayed out in the retro-peritoneum and abdominal cavity, due to its multi-lobular extensions [6], [7]. To surmount these issues, particularly with regard to spatial resolution, we used a 7 Tesla magnet, that had dedicated mouse coils, along with a transceiver coil, and started with 0.2 mm thick slices (although 0.4 mm thick slices were adequate). We also found that RARE with fat saturation was the best sequence protocol to delineate the pancreatic borders. To our knowledge, 4 other studies have used MR to image the mouse pancreas and demonstrated similar principles [19], [20], [21], [22].

He et al. used a 4.7 Tesla magnet, single RF surface transceiver coils, and 1 mm thick slices [19]. They first performed a T1-weighted spin echo with fat saturation to identify the pancreas and then switched to T2-weighted sequences to examine implanted pancreatic tumors in severe combined immunodeficiency (SCID) mice. Grimm et al. used a 7 Tesla magnet, with 0.5 mm slice thickness and a similar sequence protocol [21]. Although gadolinium contrast was used to image implanted pancreatic tumors, contrast was not given to image the pancreas. We also did not find the need to use contrast because we were able to easily differentiate the pancreas using adjacent organs as landmarks. Moore et al. used a superconducting magnet, along with antigen-specific supermagnetic nanoparticles, to track the recruitment of CD8+ T-cells to the mouse pancreas [22]. Grippo et al. performed MR microscopy in *ex vivo* fixed mouse pancreases using a very high field strength 14.1 Tesla magnet [20]. Whereas the previous studies imaged the mouse pancreas by MRI, the current study is the first to also quantify mouse pancreatic volumes.

We used our optimized protocol to compare pancreatic volumes in mice over different ages. There was a gradual growth of the mouse pancreas with advancing age, and the volumes correlated with body weight. We chose mouse ages that correspond to ages commonly used in experimental models of pancreatic disease [23], [24], [25]. We believe the information will be useful as a reference in experimental mouse pancreatic studies, although there may be some strain differences. The oldest mouse we used was a 9 month old retired breeder, which in the life-span of a mouse is analogous to a middle-aged person [26]. In comparison, Saisho et al. demonstrated that pancreatic volumes in a cohort of healthy volunteers increase from infancy to age 20 years, and after 60 years, the volumes are reduced [8]. The low variance we observed in the trend line for pancreatic volume with advancing age confirms that the imaging method can reliably detect small differences in volume, which will be beneficial in studies of pancreatic growth and recovery.

We acknowledge several limitations of our current work. We used whole-fixed mice, but future studies in ablation and recovery models of pancreatic disease will be performed in live, anesthetized mice. The Swiss-Webster mouse strain we used is generally lean, which avoids major issues of confounding intra-pancreatic fat. Nonetheless, we used a fat saturation protocol and were careful to exclude, from the tracings, any peri- or intra-pancreatic fat. We only examined males, and there may be sex differences in mouse pancreatic volumes, as shown in humans [8], [9]. We also manually traced the pancreas in each axial image, which can be time-consuming. However, our inter-observer and intra-observer variability was low. It would be ideal to develop an automated pattern recognition software to generate pancreatic volumes as reported, for example, in human heart [27].

Notwithstanding the potential for greater sophistication in MR imaging of the mouse pancreas, we believe this method in mice will benefit the wider pancreas community because mouse models are the most commonly used system to study pancreatic growth and regeneration [28]. In these conditions, changes in pancreatic volumes serve as an essential parameter of final disease outcome. A major advantage of examining mice is that, as opposed to most other animals, mice can be genetically manipulated [29]. In summary this is the first study to report that, using a high field MRI scanner and a thin-sliced, optimized sequence protocol, micro-MRI provides a powerful tool to non-invasively measure pancreatic volume in mice.
